# Supplementary material for: Cu2Se-based thermoelectric cellular architectures for efficient and durable power generation
Source: Nat Commun. 2021 Jun 10;12:3550. doi: 10.1038/s41467-021-23944-w (PMC8192747; doi:10.1038/s41467-021-23944-w)
Supplement: Supplementary file 1 — Supplementary Information [file 41467_2021_23944_MOESM1_ESM.pdf]

Cu_2Se-based thermoelectric cellular architectures for efficient and durable power generation

Seungjun Choo[1†], Faizan Ejaz[2†], Hyejin Ju[1], Fredrick Kim[1], Jungsoo Lee[1], Seong Eun Yang[1], Gyeonghun Kim[3], Hangeul Kim[1], Seungki Jo[1], Seongheon Baek[1], Soyoung Cho[1], Keonkuk Kim[1], Ju-Young Kim[1], Sangjoon Ahn[3], Han Gi Chae[1*], Beomjin Kwon[2*], and Jae Sung Son[1,4*]

[1]*Department of Materials Science and Engineering, Ulsan National Institute of Science and Technology (UNIST), Ulsan 44919, Republic of Korea.*

[2]*School for Engineering of Matter, Transport and Energy, Arizona State University, Tempe, AZ 85287, USA*

[3]*Department of Nuclear Engineering, Ulsan National Institute of Science and Technology, Ulsan 44919, Republic of Korea*

[4]*Center for Future Semiconductor Technology (FUST), Ulsan National Institute of Science and Technology (UNIST), Ulsan 44919, Republic of Korea*

[†]*These authors contributed equally to this work.*

* Correspondence to: hgchae@unist.ac.kr (H.G.C.), kwon@asu.edu (B.K.) and jsson@unist.ac.kr (J.S.S.)

This supplement contains

Supplementary Figures 1-16

Supplementary Table 1-3

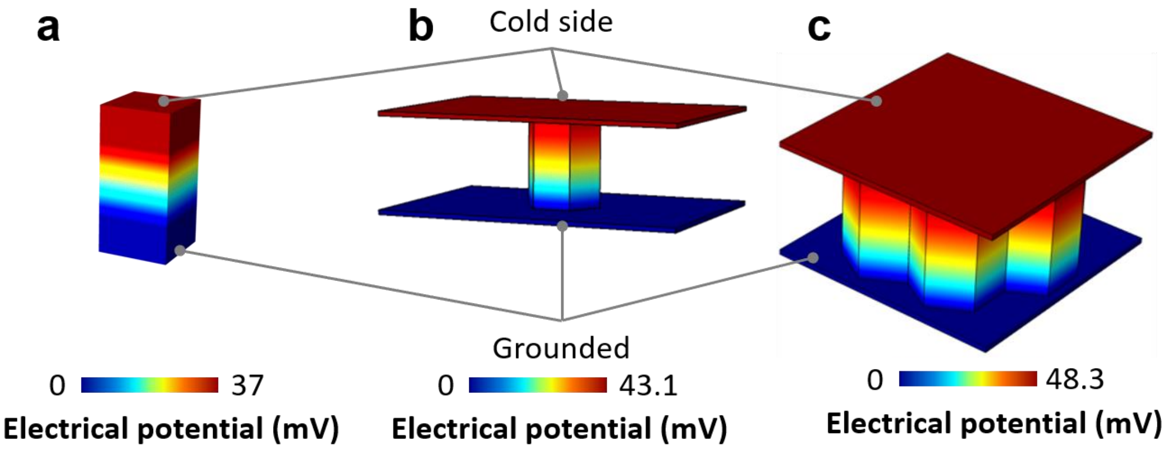

Supplementary Fig. 1 | Simulated electrical potential distributions of (**a**) cuboid-, (**b**) hollow hexagonal column-, and (**c**) honeycomb-shaped Cu_2Se TE legs.

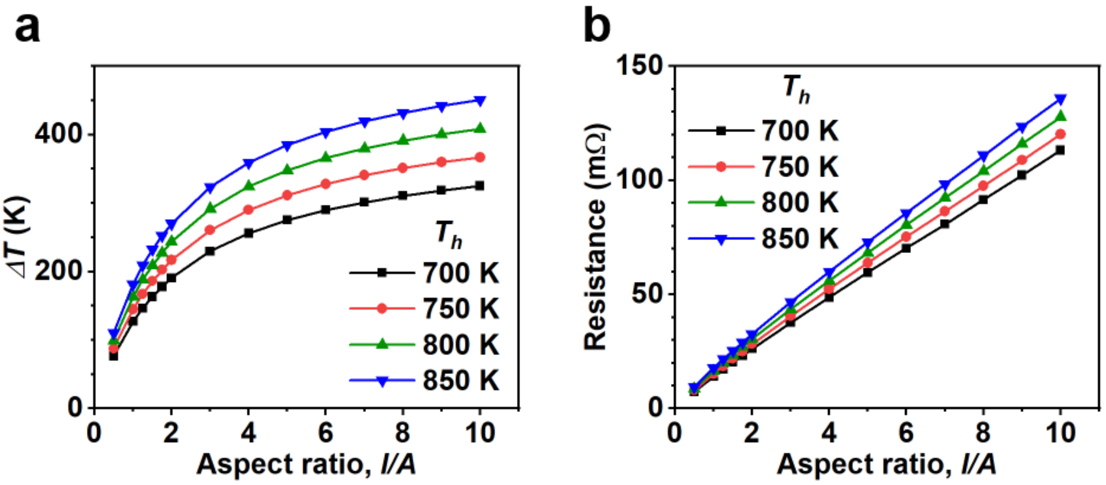

Supplementary Fig. 2 | Simulated (**a**) ΔT and (**b**) electrical resistance of a cuboid TEG as a function of aspect ratio (l/A) when the hot-side temperature is varied from 700 K to 850 K.

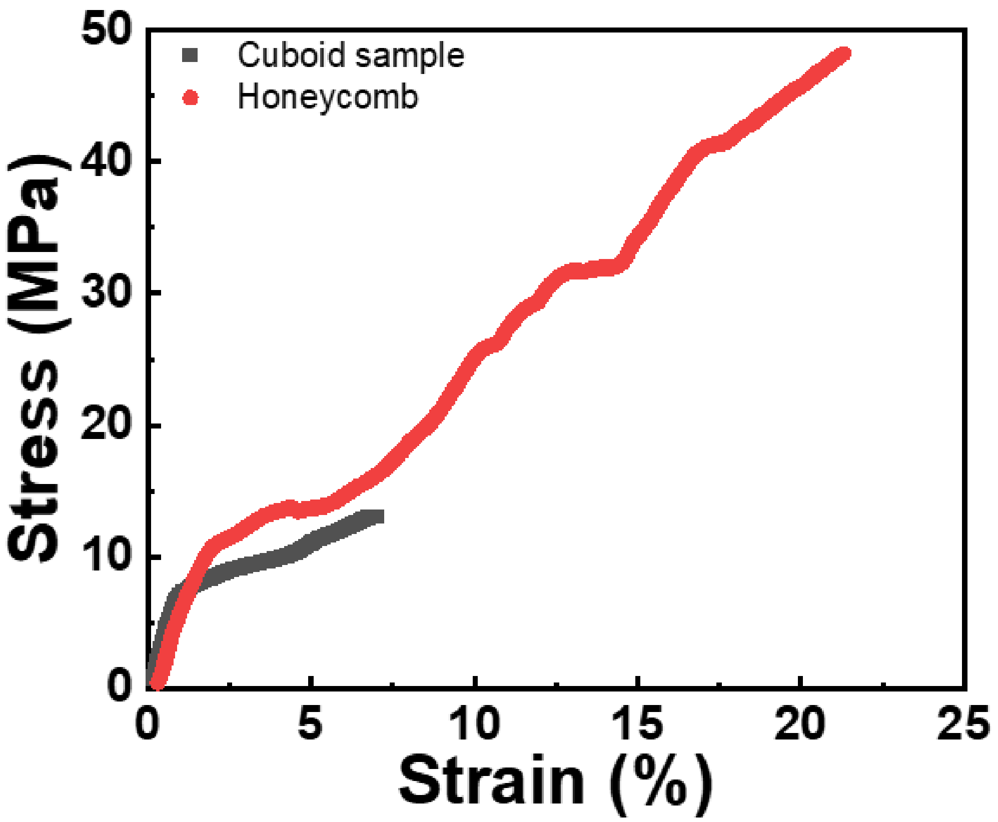

Supplementary Fig. 3 | Stress-strain curve of 3D-printed Cu_2Se cuboid and honeycomb calculated with the material's cross-sectional area by compression stress test.

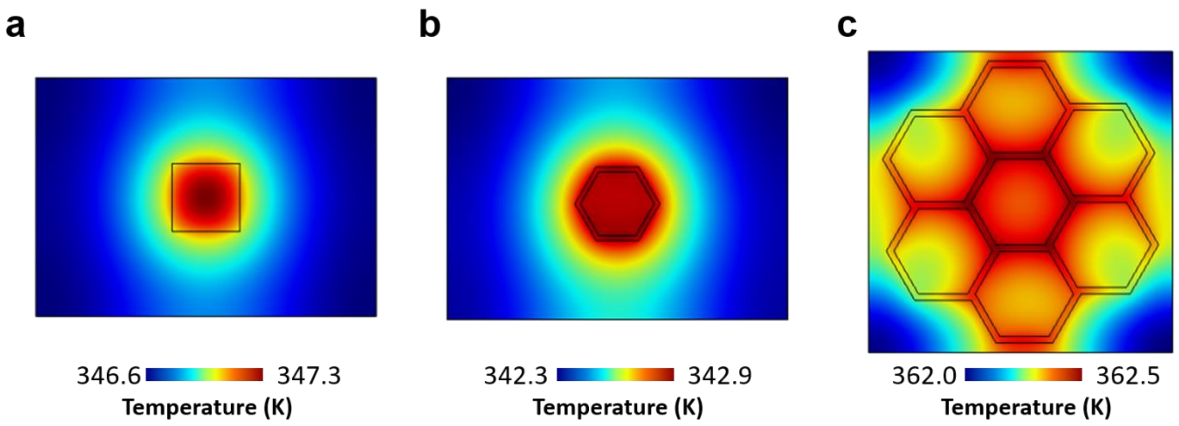

Supplementary Fig. 4 | Simulated cold-side temperature distributions of (**a**) cuboid-, (**b**) hollow hexagonal column-, and (**c**) honeycomb-shaped Cu_2Se TE legs when the hot-side temperature is 873 K.

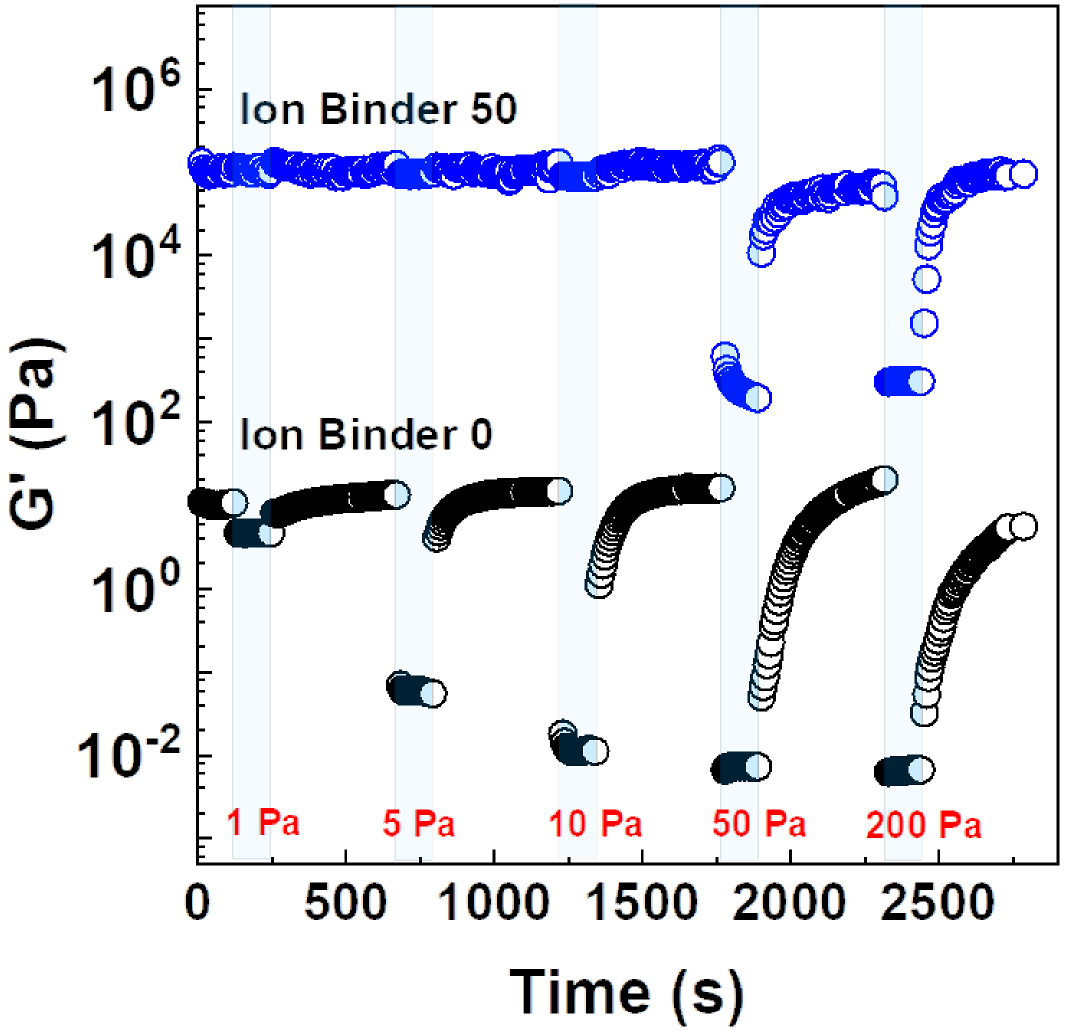

Supplementary Fig. 5 | The G' curves from the sequential 3ITTs for binder-free (black symbol) and 50 wt% Se_8^{2-} polyanion binder containing inks (blue symbol) at various shear stresses.

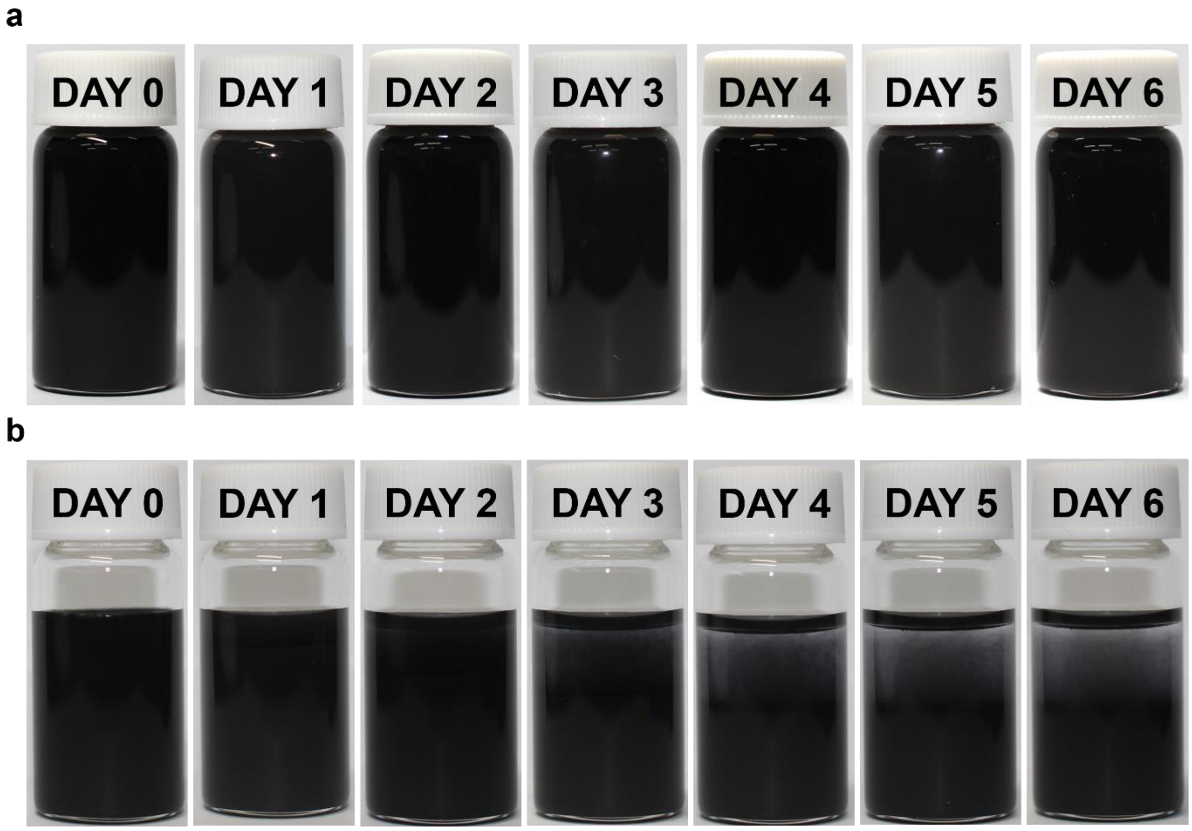

Supplementary Fig. 6 | Photographs showing the time-evolution of the dispersibility of Cu_2Se inks (**a**) with 50 wt% of Se ion binder and (**b**) without binder inks.

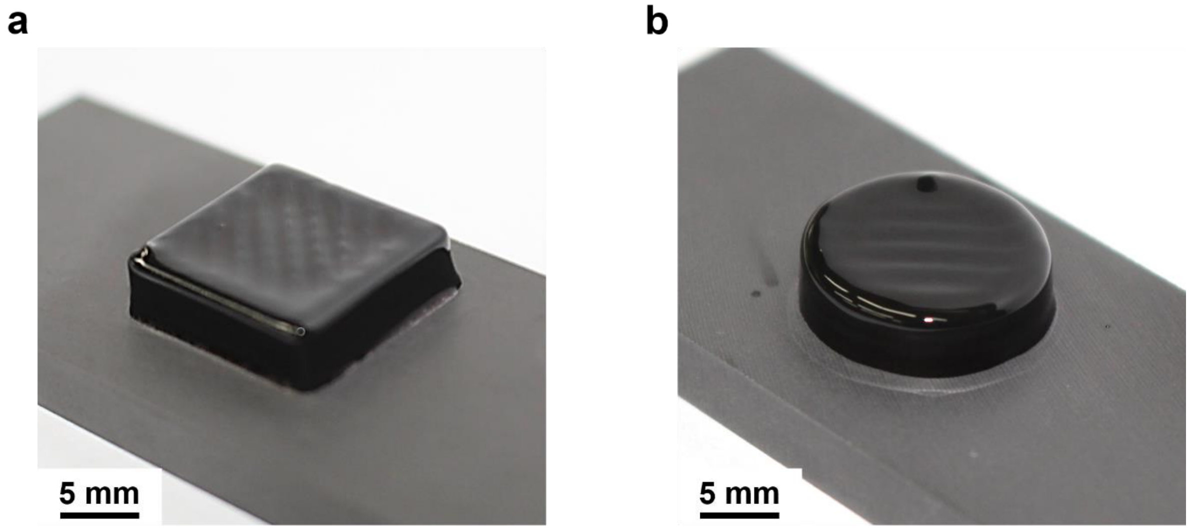

Supplementary Fig. 7 | Photographs of the 3D-printed (**a**) cuboid-and (**b**) disc-shaped Cu_2Se samples.

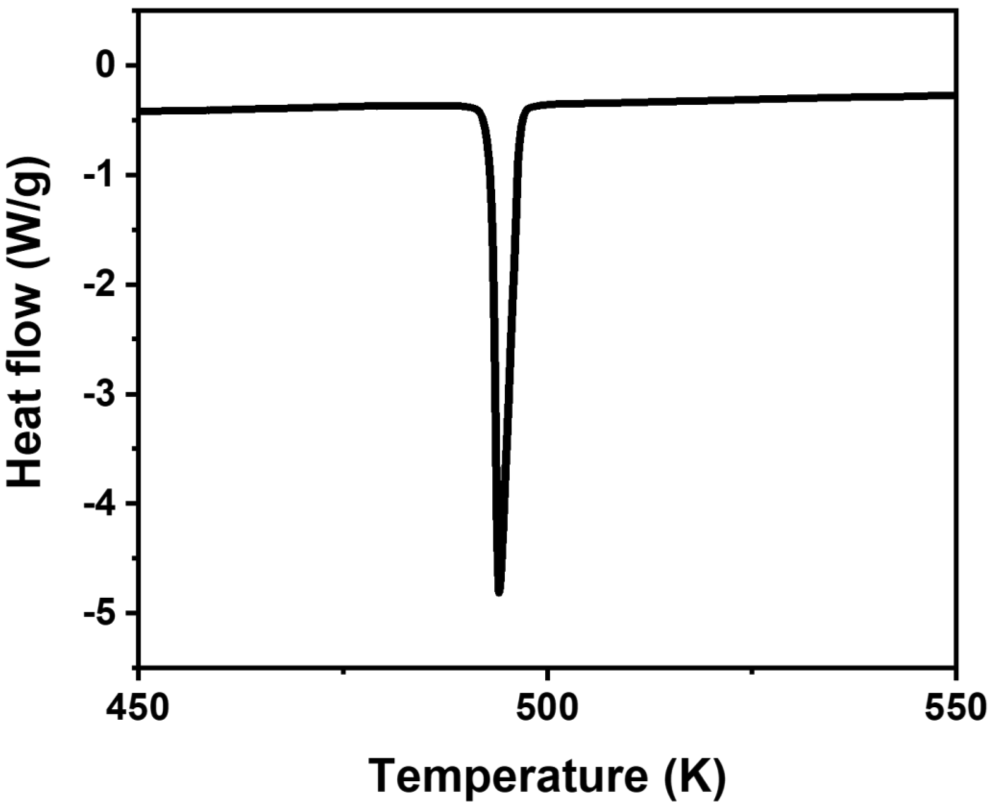

Supplementary Fig. 8 | DSC spectrum of the Cu_2Se-based ink.

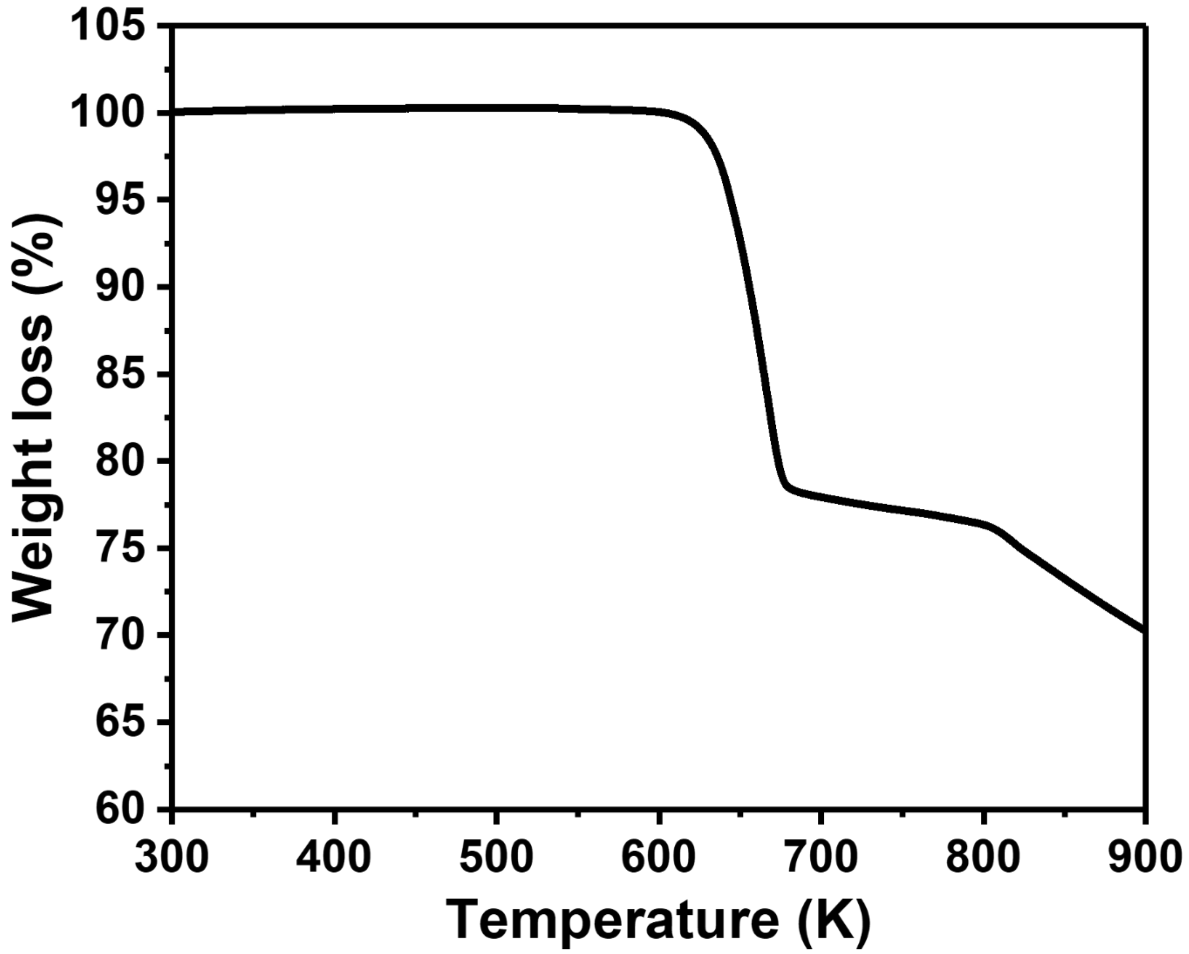

Supplementary Fig. 9 | TGA spectrum of a dried Se_8^{2-} polyanion added Cu_2Se ink.

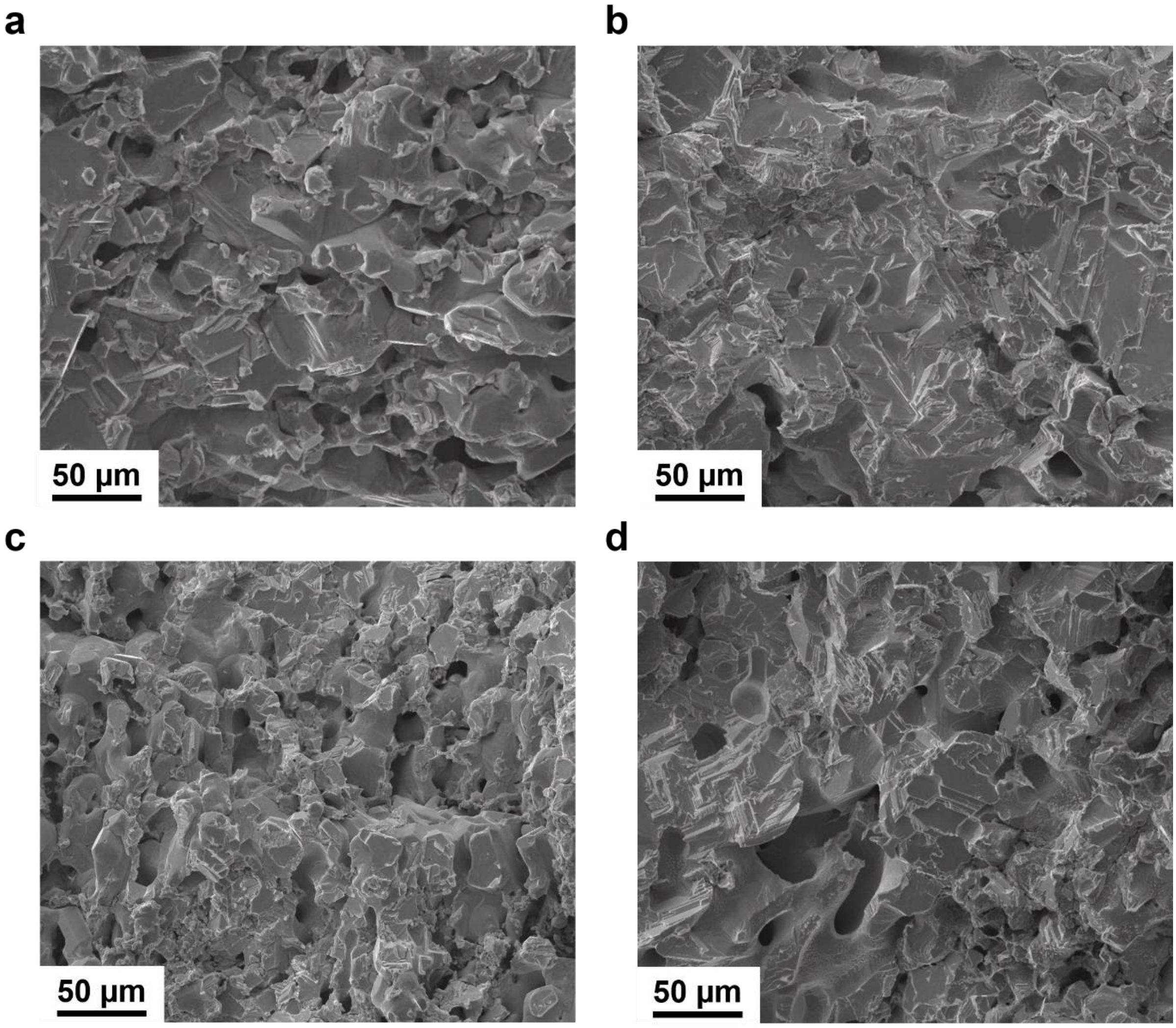

Supplementary Fig. 10 | SEM images of 3D-printed $Cu_{2-x}Se$ samples sintered for (**a**) 1 h, (**b**) 3 h, (**c**) 5 h, and (**d**) 7 h at 873 K.

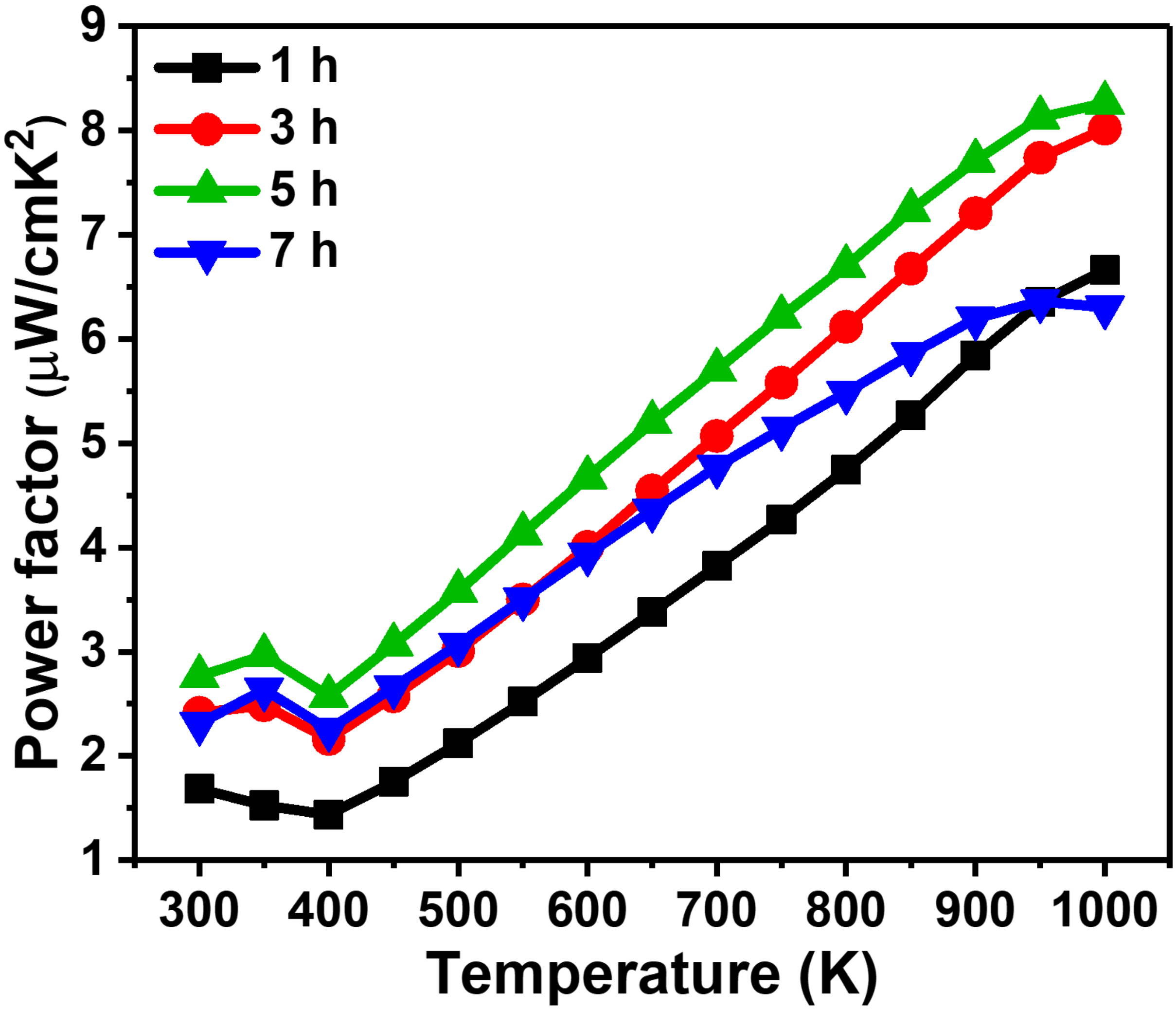

Supplementary Fig. 11 | Temperature-dependent power factors of 3D-printed Cu_2Se samples sintered at 873 K for 1, 3, 5, and 7 h.

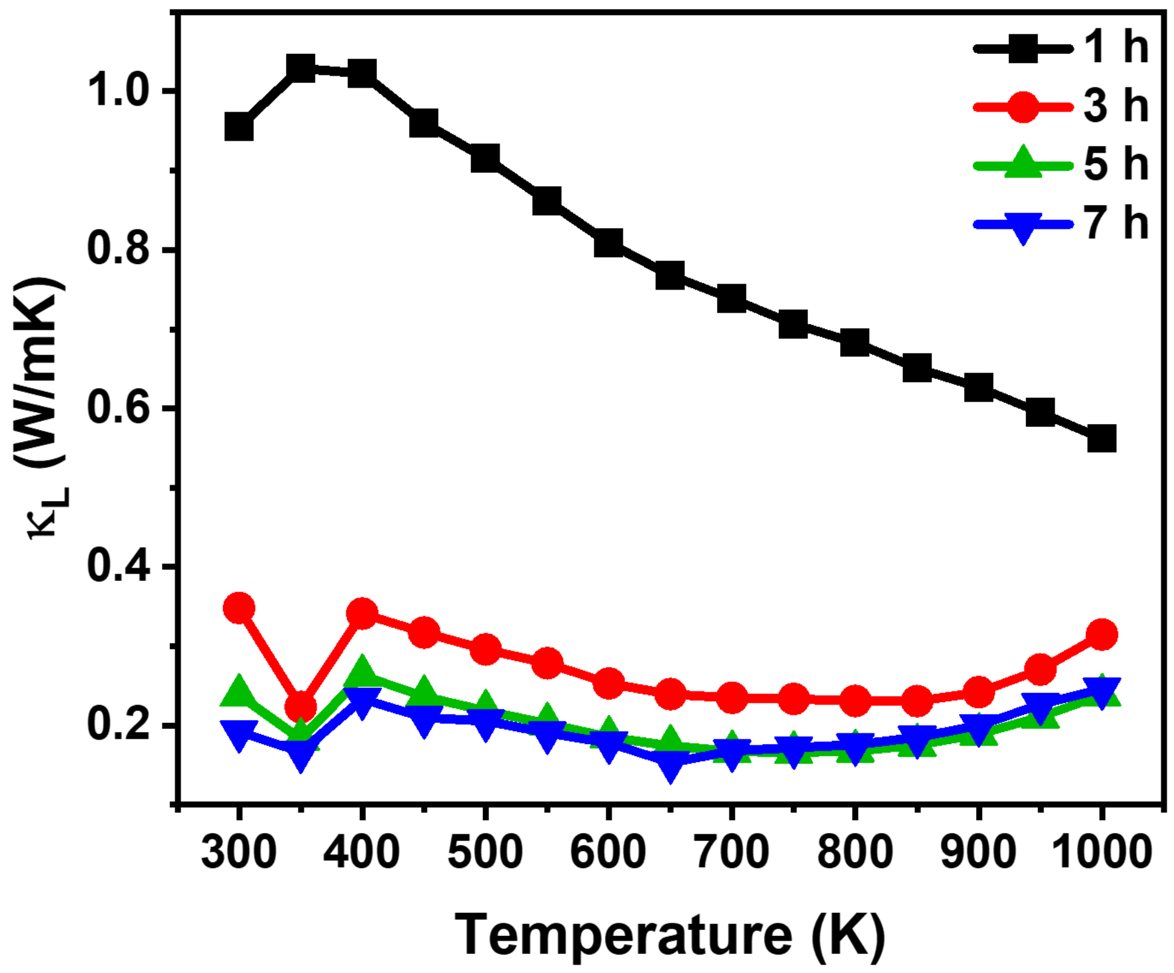

Supplementary Fig. 12 | Temperature-dependent lattice thermal conductivities of 3D printed Cu_2Se sintered at 873 K for 1, 3, 5, and 7 h.

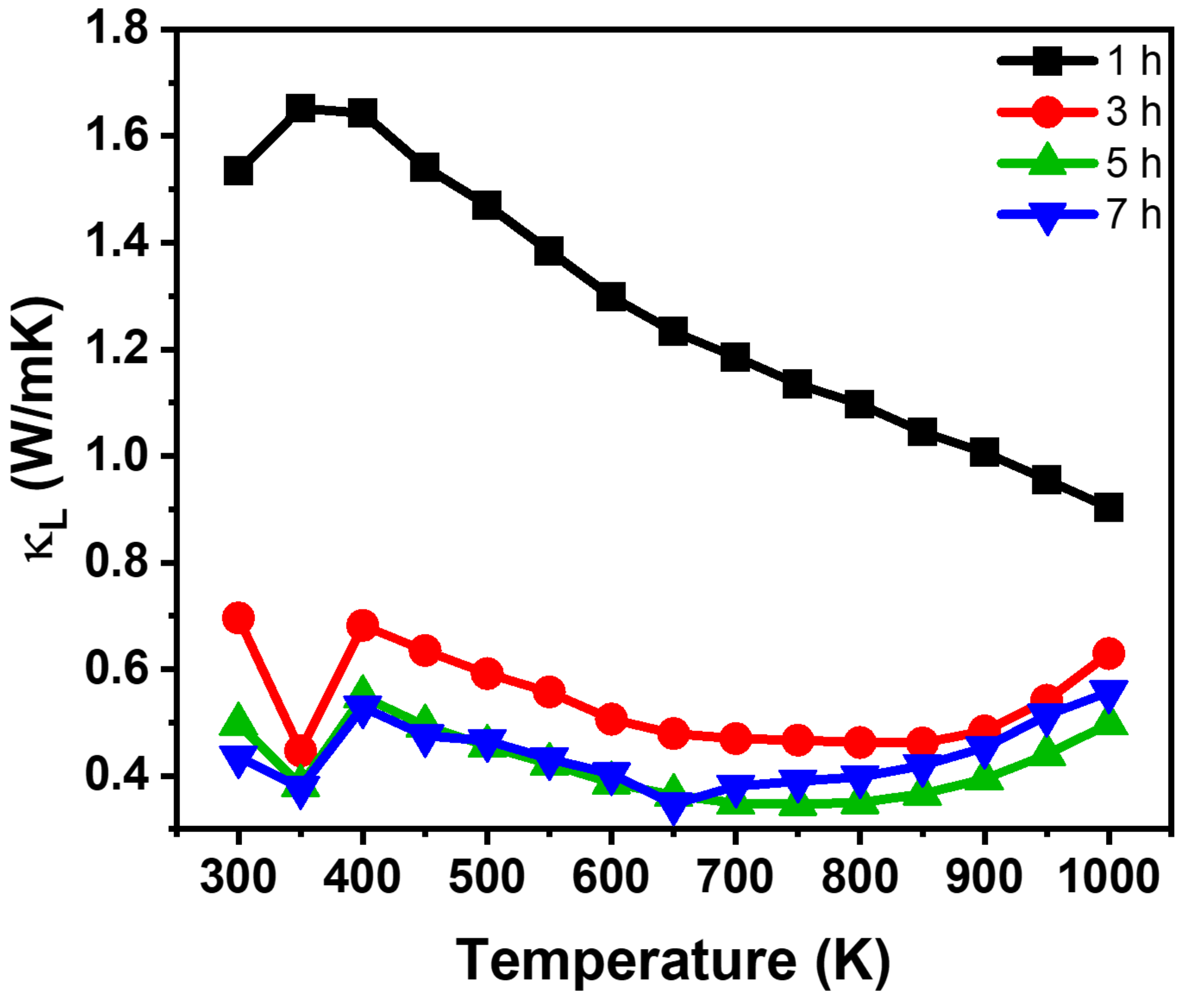

Supplementary Fig. 13 | Estimated lattice thermal conductivities of the 100% dense, 3D-printed Cu_2Se sintered at 873 K for 1, 3, 5, and 7 h using the formulation of the modified effective medium theory.

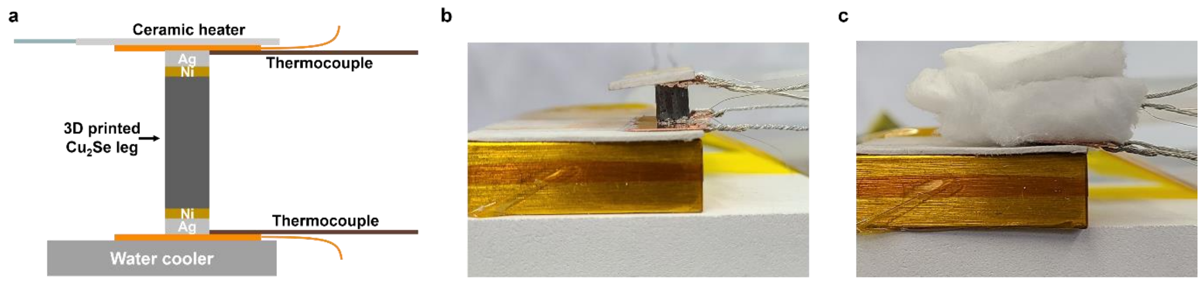

Supplementary Fig. 14 | (**a**) Scheme and photograph showing the output power measurement set-up (**b**) unveiled and veiled (**c**) with glass fabric.

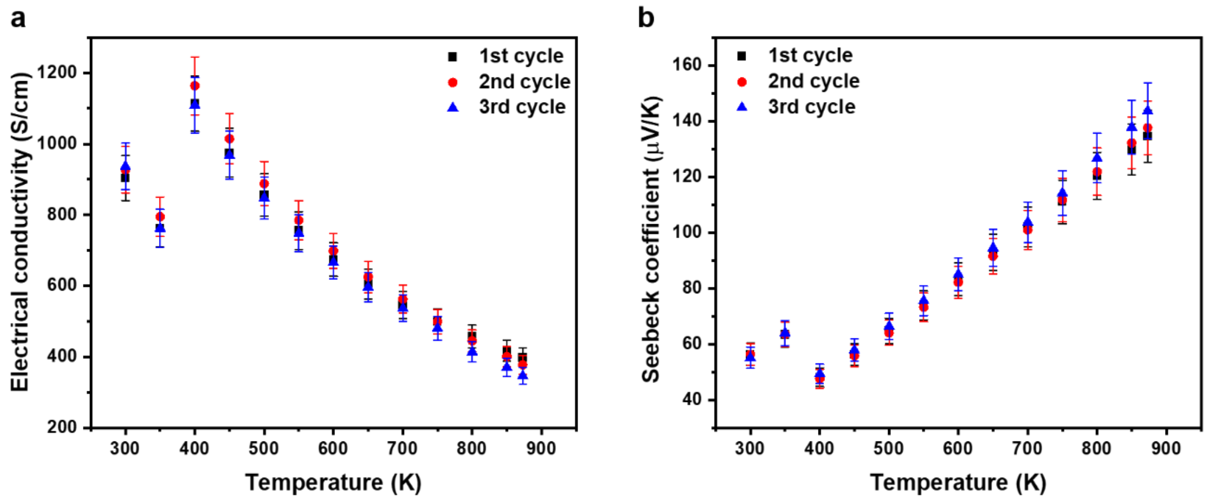

Supplementary Fig. 15 | Heat cycle performance of the 3D-printed Cu_2Se samples. Temperature-dependent (**a**) electrical conductivity, and (**b**) Seebeck coefficient. The error bars indicate the standard equipment errors of 7% for the electrical conductivity and Seebeck coefficient.

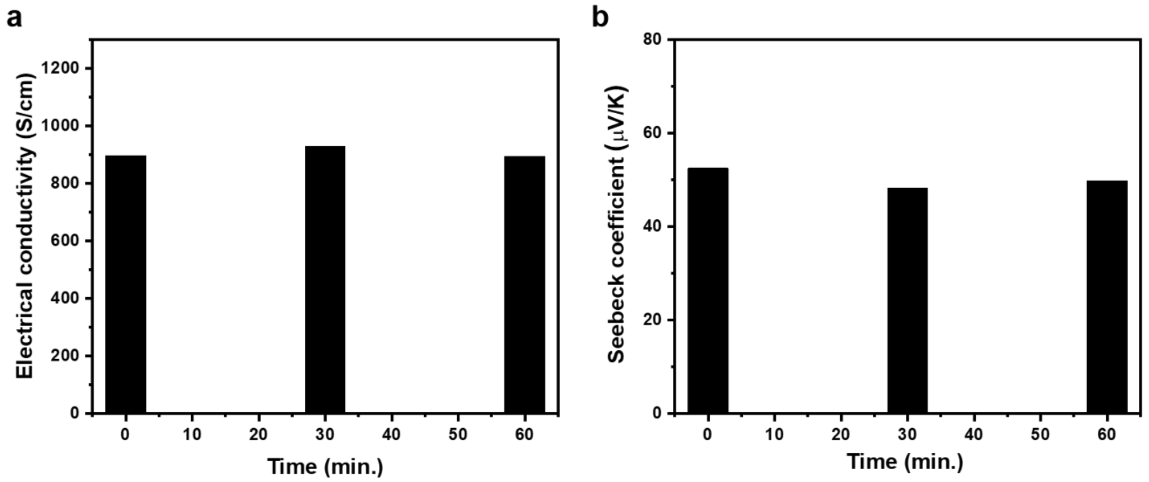

Supplementary Fig. 16 | Working stability of the 3D-printed Cu_2Se material. a, Electrical conductivity and **b**, Seebeck coefficient of the 3D-printed samples at room temperature during the heat treatment at 873 K for 1 h.

Supplementary Table 1 | Densities of the 3D-printed $Cu_{2-x}Se$ samples sintered at 873 K for 1, 3, 5, and 7 h.

	**1 h**	**3 h**	**5 h**	**7 h**
**Density (g/cm^3)**	**4.89**	**4.10**	**3.97**	**3.71**

Supplementary Table 2 | Comparison of this work with the state-of-the-art 3D-printed TE materials and modules.

**Printing method [ref]**	**Materials**	**Binder**	**TE properties**	**Remark**
Pseudo-3D printing [1]	$Cu_{2-x}S$	Sodium carboxy methylcellulose *(organic)*	*ZT*: 0.63<br>*S*: ~249.4 ɥV/K<br>κ: ~0.36 W m^{-1} K^{-1}<br>σ: ~43.3 S cm^{-1}	Molding apparatus was used (not a true 3D printing)
Selective laser meting [2]	$Bi_{0.4}Sb_{1.6}Te_3$	None	*ZT*: 1.1<br>*S*: 95 – 192 ɥV/K<br>κ: 1.6 W m^{-1} K^{-1}<br>σ: 6.05 - 11.8 S cm^{-1}	Stacking of thin layers (cubic shape) Only p type has been produced
Stereo lithography apparatus (SLA) [3]	$Bi_{0.5}Sb_{1.5}Te_3$	Photo-resin *(organic)*	*ZT*: 0.12<br>*S*: 145 – 190 ɥV/K<br>κ: 0.2 W m^{-1} K^{-1}<br>σ: ~50 S cm^{-1}	Need to remove the photo-resins by thermal decomposition (substrate-assisted bulk shape)
Pseudo-3D printing [4]	SnSe	Sodium carboxy methylcellulose *(organic)*	*ZT*: 1.7<br>*S*: 240 ɥV/K<br>κ: 0.36 W m^{-1} K^{-1}<br>σ: 1 – 4 S cm^{-1}	Molding apparatus was used (not a true 3D printing)
Screen printing [5]	TiS_2<br>(n-type)	Hexylamine *(organic)*	*ZT*: 0.22<br>*S*: 96.6 ɥV/K<br>κ: 0.69 W m^{-1} K^{-1}<br>σ: 544 S cm^{-1}	Thin layer printing of TE ink and construct cuboidal device by folding (p-type module was constructed by conductive polymer, PEDOT)
3D printing [6]	Bi_2Te_3	Poly(lactide-co-glycolide) *(organic)*	*ZT*: 0.08<br>*S*: 240 ɥV/K<br>κ: 0.25 W m^{-1} K^{-1}<br>σ: 600 S cm^{-1}	Extrusion of ink into wire shape and device construction
Aerosol jet printing [7]	Sb_2Te_3	Poly(vinylpyrrolidone) *(organic)*	*ZT*: NA<br>*S*: 83 – 105 ɥV/K<br>κ: 1 – 6 W m^{-1} K^{-1}<br>σ: 300 – 500 S cm^{-1}	Thin layer printing on curved surface
3D printing [8]	$Bi_{0.5}Sb_{1.5}Te_3$	Poly(vinylpyrrolidone) *(organic)*	*ZT*: 0.104<br>*S*: 150 ɥV/K<br>κ: 0.543 W m^{-1} K^{-1}<br>σ: 76.2 S cm^{-1}	Claimed as Direct Writing but no 3D construction of device directly by printing
Extrusion 3D printing [9]	p: $Bi_{0.4}Sb_{1.6}Te_3$<br>n: $Bi_2Sb_{2.7}Se_{0.3}$	Sb_2Te_4 chalcogenidometallate *(inorganic)*	*ZT*: 0.9 (p) / 0.6 (n)<br>*S*: 180~200 (p)/ -110~-140 (n) ɥV/K<br>κ: 0.50~0.63 W m^{-1} K^{-1}<br>σ: 550~200 (p), 500~250 (n) S cm^{-1}	1st all-inorganic ink-based 3D printed TE device

ZT: dimensionless TE figure of merit, σ: electrical conductivity (S cm^{-1}), *S*: Seebeck coefficient (μV K^{-1}), κ: thermal conductivity (W m^{-1} K^{-1}),

Supplementary Table 3 | Comparison of this work with the TE foam materials.

Ref.	Materials	Structuring purpose / benefit	Pore structure	Fabrication method	TE properties (peak *ZT*)	Remark
[10]	SiC	Enhancement of TE properties / reduction of thermal conductivity	Stochastic open pores	Macromolecule pyrogenation	*ZT*: 1.338×10^{-4}<br>*S*: ~140<br>σ: ~1.04<br>κ: ~13	Uncontrollable pore structures
[11]	SiC+Si	Enhancement of TE properties / NA	Stochastic open pores	Macromolecule pyrogenation	*S*: ~185<br>σ: 2.2<br>*PF*: 7.8	Uncontrollable pore structures
[12]	$Ca_{0.95}Sm_{0.05}MnO_3$	Using gas heat source / NA	Stochastic open pores	Sacrificial template method	*S*: ~-197	Need template or mold
[13]	CNT	Flexibility / bendable for 10000 cycles	Stochastic open pores	Rapid solvent evaporation	*ZT*: 7.6×10^{-4}<br>*S*: 32.6<br>σ: 4.02<br>κ: 0.17	
[14]	Bi_2Te_3/cellulose fiber	Flexibility / bendable for 100 cycles	Stochastic open pores	UBMS (Unbalanced magnetron sputtering) on porous cellulose fiber substrate	*ZT*: 0.38<br>*S*: ~-134<br>σ: 2100<br>κ: 0.46	Need porous substrate
[15]	PEDOT:PSS	Lightweight with flexibility / NA	Stochastic open pores	Freezing and sublimation of solvent in vacuum (aerogel)	*S*: ~19<br>σ: ~220<br>*PF* : 6.8	
[16]	PEDOT:PSS/ melamine foam	Lightweight with flexibility / NA	Stochastic open pores	Dip coating melamine foam into PEDOT:PSS solution	*ZT*: 0.007<br>*S*: 20.3<br>σ: 44.3<br>κ : 0.0905	Need porous substrate
[17]	PANI/CNTs-PDMS	Pressure sensor / dynamic pressure sensing test for 10000 cycle	Stochastic open pores	Salt-leaching method	*S* : 17.1	Need NaCl as a sacrificial template
[18]	ZnO/graphene	NA	Stochastic open pores	Salt-leaching method	N/A	Need NaCl as a sacrificial template
[19]	PVDF-PPy-MWCNT	Enhancement of TE properties / reduction of thermal conductivity	Stochastic open pores	Salt-leaching method	*ZT*: 1.5×10^{-5}<br>*S*: 19.8<br>σ: 0.055<br>κ : 0.044	Need NaCl as a sacrificial template
[20]	MWCNT/PVDF & GNP/PVDF	Enhancement of TE properties / reduction of thermal conductivity	Stochastic open pores	Used super critical carbon dioxide ($scCO_2$) as a physical blowing agent	$S_{MWCNT/PVDF}$: 6-10<br>$S_{GNP/PVDF}$: 25-58<br>$\sigma_{MWCNT/PVDF}$: ~2×10^{-4}<br>$\sigma_{GNP/PVDF}$: ~3×10^{-7}<br>$\kappa_{MWCNT/PVDF}$: ~0.1<br>$\kappa_{GNP/PVDF}$: ~0.17	Required high pressure for foam structuring
[21]	PVDF-MWCNT & PVDF-GNP	Enhancement of TE properties / reduction of thermal conductivity	Stochastic open pores	Salt-leaching method	*ZT*: ~1×10^{-3}<br>*S*: ~35<br>σ: ~1<br>κ : ~0.07	Need salt as a sacrificial template

[22]	CNT-Ag nanocomposite aerogels (CNTANAs)	Enhancement of TE properties & Lightweight / reduction of thermal conductivity	Stochastic open pores	Freezing and drying of solvent (aerogel)	*ZT*: 0.011<br>*S*: 54<br>σ: ~9.5<br>κ: ~0.075	Uncontrollable pore structures
This work	Cu_2Se	Higher energy conversion efficiency and mechanical durability / higher module power and mechanical stiffness	Periodic closed pores	3D printing	*ZT*: 1.21<br>*S*: 185.4<br>σ: 183.42<br>κ: 0.52	Controllable architectures by the 3D printing without any templates or substrates

ZT: dimensionless TE figure of merit, σ: electrical conductivity (S cm^{-1}), S: Seebeck coefficient (μV K^{-1}), κ: thermal conductivity (W m^{-1} K^{-1}), PF: power factor (μW cm^{-1} K^{-2}), T_h: hot-side temperature, T_c: cold-side temperature, *E*: elastic modulus (MPa), σ_y: yield strength (MPa), σ_f: fracture strength (MPa), CNT: carbon nano tube, PEDOT:PSS: poly(3,4-theylenedioxythiophene):poly(4-styrenesulfonate), PANI: polyaniline, PDMS: polydimethylsiloxane, PVDF-PPy-MWCNT: polyvinylidene fluoride-polypyrrole-multi-walled carbon nanotube, GNP: graphene nano-platelets, CNTANAs: CNT–Ag nanocomposite aerogels

Supplementary References

1 Burton M. R. et al. Earth abundant, non-toxic, 3D printed $Cu_{2-x}S$ with high thermoelectric figure of merit. *J. Mater. Chem. A*, **7**, 25586-25592 (2019).

2 Qiu, J. et al. 3D Printing of highly textured bulk thermoelectric materials: mechanically robust BiSbTe alloys with superior performance. *Energy & Environ. Sci.* **12**, 3106-3117 (2019).

3 He, M. et al. 3D printing fabrication of amorphous thermoelectric materials with ultralow thermal conductivity. *Small* **11**, 5889-5894 (2015).

4 Burton, M. et al. 3D printed SnSe thermoelectric generators with high figure of merit. *Adv. Energy Mater.* **9**, 26 (2019).

5 Rösch, A. et al. Fully printed origami thermoelectric generators for energy-harvesting. *npj Flex. Electron.* **5**, 1-8 (2021).

6 Peng, J. et al. 3D extruded composite thermoelectric threads for flexible energy harvesting. *Nat. Commun.* **10**, 5590 (2019).

7 Dun, C. et al. 3D printing of solution-processable 2D nanoplates and 1D nanorods for flexible thermoelectrics with ultrahigh power factor at low-medium temperatures. *Adv. Sci.* **6**, 1901788 (2019).

8 Su, N. et al. 3D-printing of shape-controllable thermoelectric devices with enhanced output performance. *Energy* **195**, 116892 (2020).

9 Kim, F. et al. 3D printing of shape-conformable thermoelectric materials using all-inorganic Bi_2Te_3-based inks. *Nat. Energy* **3**, 301-309 (2018).

10 Wei W. et al. Macrostructural influence on the thermoelectric properties of SiC ceramics. *Scr. Mater.* **57**, 1081-1084 (2007).

11 Wei W. et al. The influence of Si distribution and content on the thermoelectric properties of SiC foam ceramics. Microporous Mesoporous Mater. **112**, 521-525 (2008).

12 Reddy E. S. et al. Open porous foam oxide thermoelectric elements for hot gases and liquid environments. *Energy Convers. Manag.* **48**, 1251-1254 (2007)

13 Lee M.-H. et al. Freely shapable and 3D porous carbon nanotube foam using rapid solvent evaporation method for flexible thermoelectric power generators. *Adv. Energy Mater.* **9**, 1900914 (2019).

14 Jin Q. et al. Cellulose fiber-based hierarchical porous bismuth telluride for high-performance flexible and tailorable thermoelectrics. *ACS Appl. Mater. Interfaces* **10**, 1743-1751 (2018).

15 Gordon M. P. et al. Soft PEDOT:PSS aerogel architectures for thermoelectric applications. *J. Appl. Polym. Sci.* **134**, 44070 (2017).

16 Thongkham W. et al. Conductive nanofilm/melamine foam hybrid thermoelectric as a thermal insulator generating electricity: theoretical analysis and development. *J. Mater. Sci.* **54**, 8187-8201 (2019).

17 Wang Y. et al. 3D geometrically structured PANI/CNT-decorated polydimethylsiloxane active pressure and temperature dual-parameter sensors for man–machine interaction applications. *J. Mater. Chem. A.* **8**, 15167-15176 (2020).

18 Zhao H. et al. Conjuncted photo-thermoelectric effect in ZnO–graphene nanocomposite foam for self-powered simultaneous temperature and light sensing. *Sci. Rep.* **10**, 11864 (2020).

19 Aghelineja M. et al. Fabrication of open-cell thermoelectric polymer nanocomposites by template-assisted multi-walled carbon nanotubes coating. *Compos. Part B* **145**, 100-107 (2018).

20 Sun Y.-C. et al. Study on the thermoelectric properties of PVDF/MWCNT and PVDF/GNP composite foam. *Smart Mater. Struct.* **24**, 085034 (2015).

21 Aghelinejad M. et al. Thermoelectric nanocomposite foams using non-conducting polymers with hybrid 1D and 2D nanofillers. *Materials* **11**, 1757 (2018).

22 Sun X. et al. Thermoelectric performance of conducting aerogels based on carbon nanotube/silver nanocomposites with ultralow thermal conductivity. *RSC Adv.* **6**, 109878-109884 (2016).
