# Supplementary material for: Cu2Se-based thermoelectric cellular architectures for efficient and durable power generation
Source: Nat Commun. 2021 Jun 10;12:3550. doi: 10.1038/s41467-021-23944-w (PMC8192747; doi:10.1038/s41467-021-23944-w)
Supplement: Supplementary file 3 — Description of Additional Supplementary Files [file 41467_2021_23944_MOESM3_ESM.pdf]

Description of Additional Supplementary Files

Supplementary Movie 1: 3D printing of hollow hexagonal column of Cu_2Se
